# Public health round-up

**DOI:** 10.2471/BLT.17.010617

**Published:** 2017-06-01

**Reducing speed prevents crashes and saves lives**

Road traffic in Abidjan, Côte d'Ivoire. This year’s global road safety week campaign from 8–14 May highlighted measures to reduce the speed of road vehicles to prevent crashes and save lives. The campaign was first launched by the United Nations Road Safety Collaboration four years ago. http://www.who.int/roadsafety/week/2017

**Figure:**
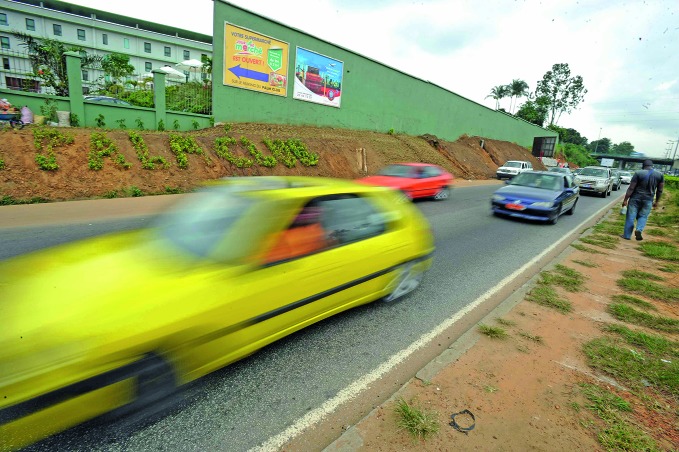
WHO/ /Andy Craggs

## Call for action on diphtheria

Supplies of diphtheria antitoxin are urgently needed worldwide for people with confirmed or suspected diphtheria disease to improve their chances of survival, according to a World Health Organization (WHO) expert group.

The Strategic Advisory Group of Experts on Immunization, which met at the end of April, called on WHO to collaborate with its partners to establish and manage a global procurement mechanism and a physical or virtual stockpile of diphtheria antitoxin.

Diphtheria is a forgotten disease in much of the world and needs global attention, the expert group said.

Progress in reducing the number of new cases of diphtheria has stalled in the last 5 years, with approximately 5000 cases reported a year, the group noted, citing data collected by WHO and the United Nations Children’s Fund.

Most diphtheria cases occur in people who have not been vaccinated at all or in those who have not had a full course of primary vaccination and booster doses, the group said.

In humans, diphtheria can cause a severe infection of the throat that can lead to respiratory failure and death. The first line of defence against diphtheria is vaccination. Suspected and confirmed cases should be treated with diphtheria antitoxin, a medicine that is included on the WHO *Model list of essential Medicines*.

The expert group also called for regulatory pathways to ensure the rapid deployment of diphtheria antitoxin.

Members of the group highlighted the need for strengthening surveillance systems to detect and investigate diphtheria cases. Better data is needed to inform vaccination schedules, prevent outbreaks and to enable a prompt response to outbreaks when they occur. Governments should report diphtheria cases caused by *C. diphtheria* and *C. ulcerans*, where laboratory capacity for confirmation is available, the expert group said.

http://www.who.int/immunization/sage/meetings/2017/April

## Tracking hepatitis progress

The first WHO *Global hepatitis report* was released in April 2017. It contains global and regional estimates on viral hepatitis for 2015 that will serve as the baseline for tracking progress on the *Global health sector strategy on viral hepatitis 2016–2021*.

The strategy calls for the elimination of viral hepatitis as a public health threat by 2030 by reducing new infections by 90% and deaths by 65%.

The report focuses on hepatitis B and C, which are responsible for 96% of all hepatitis deaths. It presents data on new infections and deaths caused by these hepatitis viruses, and the coverage of key interventions in 2015.

About 325 million people worldwide are infected with hepatitis B or C virus. Each year, 1.75 million people become infected with hepatitis C virus.

An estimated 1.34 million deaths were caused by viral hepatitis in 2015 and data from 2000 to 2015 show that these deaths have been increasing.

The report provides guidance on how to reverse this trend by scaling up interventions.

In 2015, global coverage with the three doses of hepatitis B vaccine in infancy reached 84%. These immunizations have substantially reduced hepatitis B transmission in the first 5 years of life, as reflected by the reduction in hepatitis B prevalence among children from 4.7% in the pre-vaccine era to 1.3% in 2015.

Access to affordable testing and treatment remains limited.

Only 9% of people infected with hepatitis B (22/257 million) and 20% of people infected with hepatitis C (14/71 million) have been diagnosed. While the cumulative number of persons treated for hepatitis C reached 5.5 million in 2015, only about 500 000 had received the newer, more effective and better-tolerated class of drugs called direct-acting antivirals.

In 2015 the United Nations General Assembly adopted the *2030 Agenda for Sustainable Development *calling on the international community to combat hepatitis, and in 2016 the World Health Assembly adopted the strategy on viral hepatitis with elimination as its overarching vision.

http://www.who.int/hepatitis/publications/global-hepatitis-report2017

## Mexico eliminates trachoma

Mexico has become the third country – after Morocco and Oman – to receive WHO validation for the elimination of trachoma as a public health problem.

Trachoma is the leading infectious cause of blindness worldwide. It is caused by the *Chlamydia trachomatis* bacterium and is transmitted by direct contact and flies.

The disease affects poor, isolated populations in 41 countries and had been endemic in the Mexican state of Chiapas.

In 2004, the Chiapas state health ministry launched the Trachoma Prevention and Control Programme and stepped up the delivery of a comprehensive package of WHO-recommended interventions, including surgery for advanced disease, antibiotics to clear *C. trachomatis* infection, facial cleanliness and environmental measures to reduce transmission.

In addition, the Trachoma Brigades – a group of health workers trained to combat trachoma – worked to reduce the number of cases from 1794 in 2004 to zero cases in 2016, according to data from the Chiapas Trachoma Prevention and Control Programme.

Thus, Mexico met the international criteria for elimination of trachoma as a public health problem: prevalence of less than 5% in children of 1 to 9 years of age, and less than one case of trachomatous trichiasis (inverted eyelashes) per 1000 inhabitants.

In 2012, there were only 36 cases of infection among children from 1 to 9 years of age (less than 5%) and less than 1 case of trachomatous trichiasis per 1000 inhabitants.

Mexico’s Secretary of Health, Jose Narro Robles, said that the achievement was due to “a long history of hard work and efforts by many persons, over the course of generations”.

http://www.who.int/blindness/causes/WHA51.11

**Cover photo**

A child is vaccinated against yellow fever in the Angolan city of Chitato during the 2016 yellow fever outbreak. Universal access to vaccines is one of the targets of sustainable development goal 3 on health and includes “access to quality health-care services”. This month’s theme issue focuses on the topic of quality of health care in the SDG era.

**Figure:**
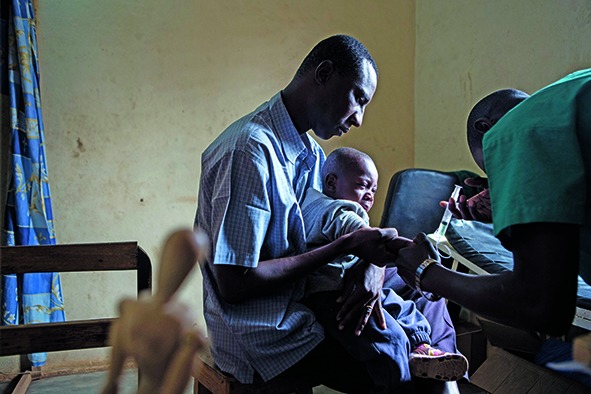
WHO/Dalia Lourenço

## Ghana, Kenya and Malawi to pilot malaria vaccine

Children in Ghana, Kenya and Malawi will receive the world’s first malaria vaccine as part of a WHO-coordinated pilot implementation programme that starts in 2018.

The RTS,S malaria vaccine has completed clinical trials, but has not yet been tested as part of a national vaccine programme – i.e. in a real-world setting. 

The pilots will assess three things: the feasibility of delivering the required our doses of RTS,S to children starting at about 5 months of age, the vaccine’s potential role for reducing childhood deaths, and its safety in the context of routine use.

The RTS,S vaccine, which is injectable, was developed to protect young children from the most deadly form of malaria caused by *Plasmodium falciparum*.

The vaccine will be assessed in the pilot programme as a complementary intervention that could be included in the core package of WHO-recommended measures for malaria prevention.

Africa bears the greatest burden of malaria worldwide. Global efforts in the last 15 years have led to a 62% reduction in malaria deaths between 2000 and 2015, but an estimated 212 million cases of malaria occurred and 429 000 people died of the disease in 2015, most of them young children in Africa.

“The prospect of a malaria vaccine is great news. Information gathered in the pilot will help us make decisions on the wider use of this vaccine,” said Dr Matshidiso Moeti, WHO Regional Director for Africa. “Combined with existing malaria interventions, such a vaccine would have the potential to save tens of thousands of lives in Africa.”

RTS,S was developed by GlaxoSmithKline (GSK) and is the first malaria vaccine to have successfully completed a Phase III clinical trial. The trial was conducted between 2009 and 2014 by a partnership involving GSK, the PATH Malaria Vaccine Initiative with support from the Bill & Melinda Gates Foundation and a network of research sites in Burkina Faso, Gabon, Ghana, Kenya, Malawi, Mozambique and United Republic of Tanzania.

The participation of Ghana, Kenya and Malawi in the implementation pilot was announced on World Malaria Day on 25 April with the launch of a new WHO report entitled *Malaria prevention works: let’s close the gap*.

http://www.who.int/malaria/publications/atoz/malaria-prevention-works

## World report on health policy and systems research

A new report describes the evolution of health policy and systems research over the last 20 years, including data on publications in this field, funding trends, global collaborations and a review of low- and middle-income countries’ capacity to conduct this type of research.

The *World Report on health policy and systems research*, released in April, provides insight into how countries can take a multidisciplinary and systems approach to make progress towards universal health coverage and the sustainable development goals.

The report was launched by the Alliance for Health Policy and Systems Research, an international collaboration hosted by WHO.

http://www.who.int/alliance-hpsr/news/2017/worldreport-hpsr

**Looking ahead**

1 July – WHO’s new Director-General takes office

1-3 November – World Hepatitis Summit 2017. São Paulo, Brazil

16-17 November – Global Ministerial Conference on tuberculosis. Moscow, Russian Federation

